# The Action of Caffeine, Morphine, Atropine, Ergot, and Digitalis on the Arterial Blood Pressure

**Published:** 1891-06-20

**Authors:** 


### THERAPEUTICS.

### THE ACTION OF CAFFEINE, MORPHINE, ATROPINE, ERGOT, AND DIGITALIS ON THE ARTERIAL BLOOD PRESSURE.

Dr. Sophie Frenkel has conducted by means of the sphygmograph of Basch, a series of clinical observations with these remedies, in reference to their effects on the blood pressure in man. This instrument of Von Basch is the only form of manometer by means of which blood pressure can be measured in human beings. Even this sphygmomanometer can only yield relative, not absolute results. Caffeine was given by the mouth as well as subcutaneously. By the mouth in daily doses of from 7½ to 12 grains administered to cases of heart disease with failure of compensation, it tended to increase the arterial pressure, removed the symptoms of loss of compensation, very much improved the subjective symptoms, and increased the amount of urine passed. The elevation of arterial pressure produced by caffeine is, as a rule, slight, reaching its maximum intensity on the second or third day, and after remaining stationary for one or two days, gradually declines, although the remedy be continued. The diuretic action does not appear to depend on the rise in blood pressure, for it may precede it and frequently outlasts it. There is no cumulative action. The pulse was hardly affected. In eight cases the effect of one large dose subcutaneously administered, was observed, and increased arterial pressure was noted almost immediately. The author recommends this method in the treatment of collapse in cardiac subjects and various other pathological conditions. Morphine in subcutaneous doses of $\frac{1}{6}$ to $\frac{1}{2}$ grain in fifteen cases sometimes produces a slight increase in arterial pressure, sometimes is without influence upon it, the pulse being but slightly modified. The patients were mostly suffering from chronic nervous diseases, only one having an abnormal heart. As morphine is frequently used to control hæmoptysis under the impression that arterial pressure is lessened, these results are not without practical interest.

Sulphate of atropine injected subcutaneously in doses of from about $\frac{1}{120}$ to $\frac{1}{60}$ grains, produced a marked increase in blood pressure, usually in about twenty minutes or an hour, and lasting for about two hours. The pulse was sometimes accelerated, the diuretic effect *nil*. The dryness of the mouth, and various other unpleasant effects induced, render the use of this drug undesirable in cardiac cases. With ergotin the blood pressure was raised to about the ame extent as from atropine, and, like it, produces its effect for about two hours, the pulse being usually considerably slowed. Digitalis gave a marked rise in blood pressure, and the observations simply confirmed the generally received views as to its action.*

* Dent. Archiv. f. Klin. Med., Hft. 6. p. 542; Gaz. Méd. de Par., Oct. 11, 1890.

